# Respiratory and sleep disorders in mucopolysaccharidosis

**DOI:** 10.1007/s10545-012-9555-1

**Published:** 2012-11-15

**Authors:** Kenneth I. Berger, Simone C. Fagondes, Roberto Giugliani, Karen A. Hardy, Kuo Sheng Lee, Ciarán McArdle, Maurizio Scarpa, Martin J. Tobin, Susan A. Ward, David M. Rapoport

**Affiliations:** 1 Department Medicine, Physiology and Neuroscience, André Cournand Pulmonary Physiology Laboratory, Bellevue Hospital, New York University School of Medicine, New York, NY USA; 2 Department Pulmonary Medicine, Hospital de Clinicas de Porto Alegre, Porto Alegre, Brazil; 3 Department Genetics/UFRGS, Medical Genetics Service/HCPA and INAGEMP, Porto Alegre, Brazil; 4 Children’s Hospital & Research Center Oakland, Oakland, CA USA; 5 Mackay Memorial Hospital, Taipei, Taiwan; 6 Birmingham Childrens Hospital, Birmingham, UK; 7 Department Pediatrics, University of Padua, Padua, Italy; 8 Hines VA Hospital, Hines, IL USA; 9 Human Bio-Energetics Research Centre, Crickhowell, UK; 10 Department of Medicine, New York University School of Medicine, New York, NY USA; 11 Department of Medicine, Physiology and Neuroscience, New York University School of Medicine, 550 First Ave, Room RR108, New York, NY 10016 USA

## Abstract

MPS encompasses a group of rare lysosomal storage disorders that are associated with the accumulation of glycosaminoglycans (GAG) in organs and tissues. This accumulation can lead to the progressive development of a variety of clinical manifestations. Ear, nose, throat (ENT) and respiratory problems are very common in patients with MPS and are often among the first symptoms to appear. Typical features of MPS include upper and lower airway obstruction and restrictive pulmonary disease, which can lead to chronic rhinosinusitis or chronic ear infections, recurrent upper and lower respiratory tract infections, obstructive sleep apnoea, impaired exercise tolerance, and respiratory failure. This review provides a detailed overview of the ENT and respiratory manifestations that can occur in patients with MPS and discusses the issues related to their evaluation and management.

**Electronic supplementary material:**

The online version of this article (doi:10.1007/s10545-012-9555-1) contains supplementary material, which is available to authorized users.

## MPS disease overview

The mucopolysaccharidoses (MPS) are rare inherited metabolic diseases associated with deficiencies in enzymes involved in the degradation of glycosaminoglycans (GAG) (Muenzer 2011). Progressive accumulation of GAG in multiple tissues and organs (Table 1) lead to an array of manifestations that worsen with age, ultimately resulting in severe morbidity and premature death (Muenzer 2011). Most patients share a typical appearance characterized by coarsened facial features, reduced height, and skeletal abnormalities. Other manifestations include impaired vision and hearing, hepatosplenomegaly, cardiovascular disease, spinal cord compression, and ear-nose-throat (ENT) and respiratory problems. Cognitive impairment is the main feature in MPS III and may occur in MPS I, II and VII. Although many similarities exist, the spectrum and severity of disease manifestations vary between and within the different MPS types.

**Table 1:** Overview of types of mucopolysaccharidosis (MPS) and glycosaminoglycans that accumulate in each of these MPS types

MPS		Dermatan sulfate (DS)	Heparan sulfate (HS)	Keratan sulfate (KS)	Chondroitin sulfate (CS)
I (H, HS, S)	Hurler, Hurler-Scheie, Scheie	+	+		
II	Hunter	+	+		
III	Sanfilippo		+		
IVa	Morquio			+	+
IVb				+	
VI	Maroteaux-Lamy	+			+
VII	Sly	+	+		+

This review addresses ENT and respiratory disorders occurring in MPS, and discusses their evaluation and management.

## Physiology of respiration and impact of sleep

Respiration results from the interaction of pathways under cerebral control. The brain controls the synchronous movement of the diaphragm, ribs and abdomen; modulating influences are exerted by feedback from arterial blood gas tensions, pH and pulmonary-mechanical factors. The brain also controls phasic coordination of upper airway (UA) muscles, which interact with anatomic factors to determine the UA resistance. Coordinated activity of the chest wall and UA muscles is required to achieve normal ventilation. Changes in UA and diaphragmatic control occur with sleep onset, and can thus disrupt ventilation.

## ENT and respiratory disorders in MPS

**Fig. 1:**
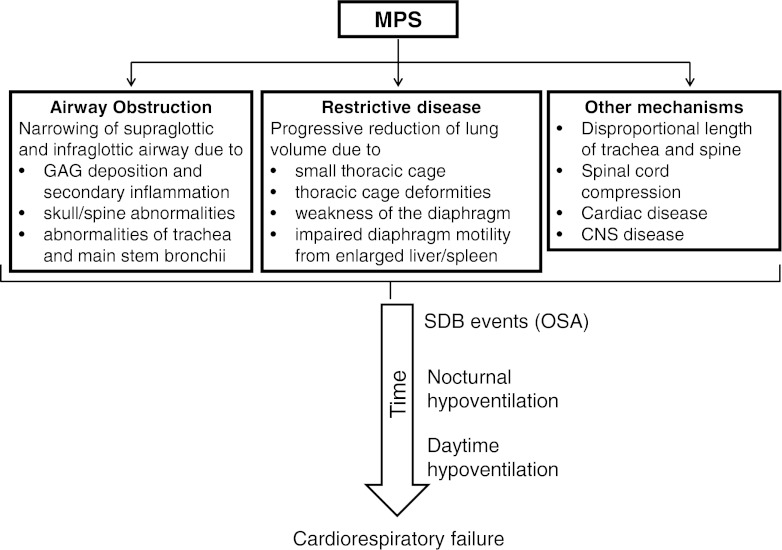
Mechanisms that predispose to sleep disordered breathing in patients with mucopolysaccharidosis (MPS)

Respiratory disorders occur in all MPS types (John et al 2011; Leighton et al 2001; Lin et al 2010; Muhlebach et al 2011; Nashed et al 2009; Santamaria et al 2007; Semenza and Pyeritz 1988) (Table 2). However, due to low patient numbers no conclusions can be made regarding the prevalence and severity of these problems in each MPS type. The ENT and respiratory disorders can be divided into airway abnormalities, alterations in respiratory mechanics, and effects of sleep (Fig. 1 and Table 3).

**Table 2:** Key respiratory manifestations in the different mucopolysaccharidosis (MPS) types. Adapted from Muhlebach et al 2011, with permission from Elsevier Ltd

MPS	Upper airway obstruction	Lower airway obstruction	Restrictive lung disease
I	+++	+++	+++
II	+++	+++	++
III	Minimal	Minimal	Minimal
IV	++	+	+++
VI	+++	+++	++
VII	+++	+++	++

**Table 3:** Manifestations of mucopolysaccharidosis (MPS) that may affect respiratory function and their prevalence in a study including 21 patients with MPS I, II, IV, VI, and VII. Adapted from Semenza et al (Semenza and Pyeritz 1988), with permission from Williams & Wilkins

	% of patients (*N* = 21)
Airway narrowing	
Hypertrophy of tonsils/adenoids	67
Macroglossia	57
Nasopharyngeal obstruction	86
Supraglottic narrowing	92
Infraglottic narrowing	62
Pulmonary disease	
Obstructive defect	71
Focal atelectasis	35
Recurrent pneumonia	40
Interstitial markings	24
Arterial hypoxemia awake	57
Arterial hypoxemia asleep	100
Sleep apnoea	
Central	0
Obstructive	89
Thoracolumbar spine deformity	
Scoliosis	67
Thoracic hyperkyphosis	62
Lumbar hyperlordosis	38
Thoracolumbar gibbus	57
Cervical spine involvement	
Cervical spine subluxation	68
Odontoid hypoplasia	69
Cord compression	63

### Airway abnormalities

Supraglottic manifestations are common in MPS and develop due to cranial and spinal abnormalities (e.g., flattened nasal bridge, short neck, high epiglottis, mandibular abnormalities, abnormal cervical vertebrae) and GAG deposition in the mouth, nose and throat (Leboulanger et al 2011; Leighton et al 2001; Myer 1991; Shih et al 2002). Oral manifestations include gingival hyperplasia, mucosal oedema, mucoid secretions, and impaired opening of the mouth. Chronic rhinosinusitis and chronic otitis media may occur and produce hearing impairment (John et al 2011; Muhlebach et al 2011), and the rhinosinusitis can contribute to UA obstruction during sleep.

GAG storage can also cause distension of the tongue, adenoids and/or tonsils with formation of collapsible, space-occupying lesions in pharyngolaryngeal walls (Simmons et al 2005). In extreme cases, excessive tissue at the arytenoid cartilages and aryepiglottic folds can prolapse into the laryngeal inlet causing stridor and airway compromise (Simmons et al 2005). Obstruction is often worsened by presence of thickened secretions throughout the upper and lower respiratory tracts (Leighton et al 2001). ENT manifestations are among the first disease-specific manifestations to appear, may trigger diagnosis of MPS (Muhlebach et al 2011; Wold et al 2010), and tend to progress with age.

Airway abnormalities and airway collapse may also occur in infraglottic airways, including the trachea and central airways. Tracheobronchomalacia may develop secondary to GAG deposition in the tracheobronchial cartilage (Nagano et al 2007; Pelley et al 2007; Shih et al 2002; Sims and Kempiners 2007). Alternatively, tracheal collapse can occur due to decreased tracheal traction from decreased lung volume (Heinzer et al 2005), although this has not been specifically shown in MPS. MPS IV patients may develop airway occlusion upon neck flexion. These patients assume a “sniff position” (extension of the neck to increase airway patency) during wakefulness and may sleep in the prone position. Clinical manifestations include dyspnoea, difficulty clearing secretions, cough, wheezing, and recurrent bronchitis or pneumonia (John et al 2011; Leighton et al 2001; Muhlebach et al 2011; Murgu and Colt 2006; Pelley et al 2007). Another study also suggested alveolar and interstitial pulmonary involvement by GAG deposition (Semenza and Pyeritz 1988). Airway obstruction and parenchymal abnormality have potential to impair pulmonary O_2_ uptake and CO_2_ excretion.

### Alteration in respiratory mechanics

Multiple abnormalities in MPS patients can reduce ventilatory capacity, manifesting as reduction in vital capacity (VC). Kyphoscoliosis and pectus carinatum are common and alter chest wall shape and structure. Diaphragm excursion may be compromised by liver and spleen enlargement (Buhain et al 1975; Giugliani et al 2007; Leighton et al 2001). Diaphragmatic weakness may result from spinal cord compression above the phrenic nerve origin (C3-C5), but there is currently no evidence for this hypothesis.

Pulmonary function can also be affected by the characteristic short stature and skeletal dysplasia. In MPS disorders associated with short stature (e.g., MPS VI), patient height is the primary determinant of VC and improved pulmonary function during enzyme replacement therapy (ERT) is frequently associated with growth and increased stature (Swiedler et al 2005; Harmatz et al 2010).

The combined effect of these alterations in respiratory mechanics coupled with airway abnormalities may lead to respiratory failure.

### Effects of sleep

Ventilatory compromise is exacerbated by normal sleep mechanisms that increase UA collapsibility, thus magnifying any impairment to generation of inspiratory effort. The effects of sleep are particularly evident during REM sleep, which causes loss of tone in accessory muscles of respiration and reduction in ventilatory CO_2_ chemosensitivity (Dempsey et al 2010). Due to the changes that occur with sleep onset, ventilatory compromise often first manifests as sleep disordered breathing (SDB) and unexplained hypoxemia during sleep.

**Fig. 2:**
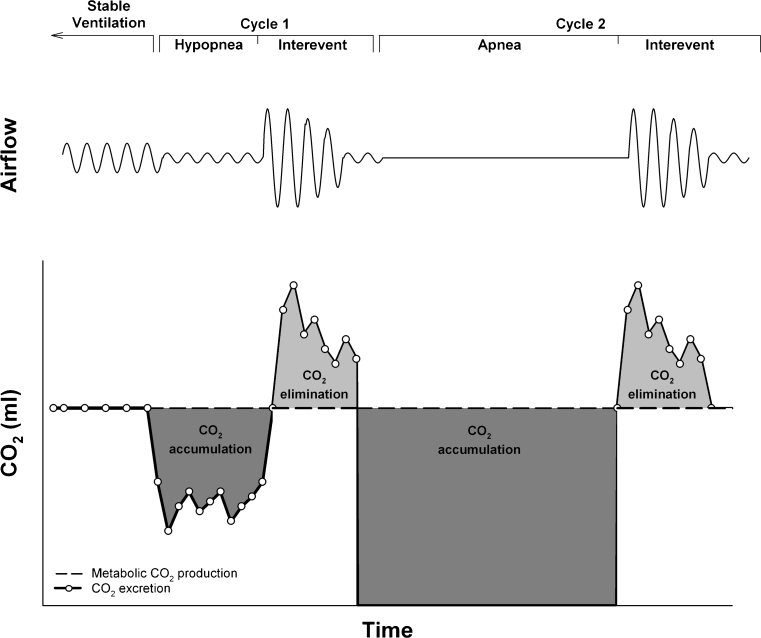
Impaired compensation for sleep disordered breathing, leading to hypoventilation during sleep. Reproduced from Berger et al (Berger et al 2000), with permission from the American Physiology Society

SDB occurs in >80 % of MPS patients (John et al 2011; Leighton et al 2001; Semenza and Pyeritz 1988). It can be categorized as either obstructive sleep apnoea (OSA) or sustained hypoventilation. OSA occurs from an interaction between abnormalities of the skull and GAG deposition in the UA and perhaps from remote effects of thoracic deformation causing reduced lung volumes. OSA can lead to daytime sleepiness and long-term cardiovascular sequellae and often presents as loud and disruptive snoring. Sleep studies demonstrate recurrent short episodes (<90 s) of either reduced or absent ventilation due to UA obstruction. In more advanced stages, compensation for CO_2_ retention during individual SDB events may be impaired by reduction of VC and inability to increase ventilation during inter-event intervals (Fig. 2) (Berger et al 2000). These patients exhibit progressive CO_2_ retention throughout the night, i.e., nocturnal hypoventilation (Mellies et al 2003; Ragette et al 2002).

A restrictive abnormality and/or diaphragmatic weakness may also cause sustained periods of hypoventilation. Sleep studies demonstrate prolonged periods (several minutes) of arterial O_2_ desaturation consequent to hypoventilation (Leighton et al 2001). Reduced VC, which is influenced by respiratory muscle strength, may be a good predictor of SDB severity (Ragette et al 2002). Whereas patients with VC <60 % of predicted have a predisposition to SDB, those with a VC <40 % of predicted are prone to sustained hypoventilation and CO_2_ retention.

If improperly managed, nocturnal hypoventilation may lead to pulmonary hypertension, cor pulmonale and eventually respiratory failure (John et al 2011; Semenza and Pyeritz 1988), a common cause of death in MPS (Leighton et al 2001).

### Other potential mechanisms

Tracheal distortion is characteristic for MPS and may reflect disproportionate length of the trachea relative to the shortened spinal length (Walker et al 2003). In combination with laxity of tracheal tissue, it can cause airway collapse (Walker et al 2003). Cervical or lumbar spinal cord lesions may weaken expiratory muscles, impair cough and reduce secretion clearance, predisposing patients to recurrent pneumonia. In patients with cardiac abnormalities or chronic respiratory failure, nocturnal shift in leg fluid volume may cause UA oedema. Furthermore, the respiratory arousal threshold (i.e., the ease with which an individual wakes up in response to respiratory stimuli) may be modified due to brain modifications. However, these issues remain to be investigated.

## Assessment of respiratory function in MPS patients

### Evaluation of UA anatomy

**Fig. 3:**
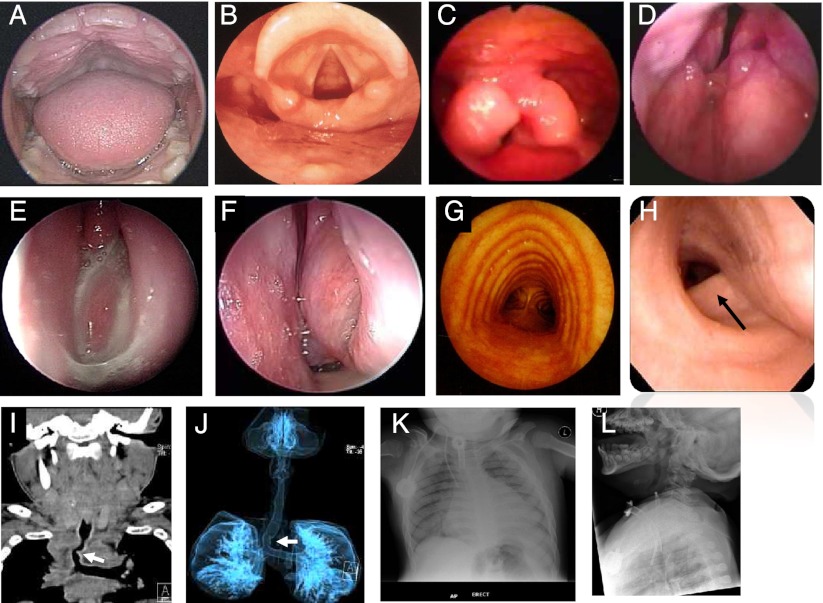
**a** Oropharyngeal image from a patient with mucopolysaccharidosis (MPS) II showing macroglossia and gingival hyperplasia; **b** normal lyaryngoscopic image; **c**-**d** laryngoscopic images from a patient with MPS II showing a thick laryngeal wall, tonsillar hyperplasia, and redundant arytenoid mucosa prolapse into laryngeal inlet; **e**-**f** nasal endoscopic images showing inflammation and abnormal mucous secretion in a patient with MPS VI; **g** normal bronchoscopic image (Canani et al 2003); **h**-**j** bronchoscopic image and CT scans from a patient with MPS IV showing tracheal deformity and narrowing; **k**-**l** typical frontal and lateral plain X-rays of the chest of a child with MPS II showing skeletal abnormalities and the presence of a tracheostomy and possibly infective changes in the lungs (hazy shadow in the right lower zone of the frontal image)

Classification systems for grading mouth and UA abnormalities in MPS include a modified Mallampati classification system based on visibility of the tonsils, pillars, uvula and soft palate and a classification system for grading tonsillar hypertrophy (Friedman et al 1999). Computed tomography (CT) of retropalatal and retroglossal spaces can demonstrate reduction in UA volume in MPS patients (Santamaria et al 2007). Multi-detector CT imaging can also evaluate airway size, airway structure and the lung parenchyma (Ingelmo et al 2011) (Fig. 3i and j).

Dynamic changes in the airway during respiration can be evaluated using flexible endoscopy (Fig. 3a, b, c, d, e, f, g and h). Fiberoptic examination is preferably done in a sitting position, which requires less ventilatory support than the supine position. Pharyngeal and laryngeal abnormalities can be classified based on the level of mucosal alteration (personal communication Dr. A. Keilmann). Grading systems based on adenoid hypertrophy and obstruction of the choanal opening and tubal ostium exist to evaluate nasal cavity obstruction (Cassano et al 2003; Josephson et al 2011; Parikh et al 2006).

Plain roentgenograms of the chest, spine and soft tissue of the neck may demonstrate airway obstruction, atelectasis, skeletal changes that cause restrictive disease and/or spinal cord compression (Fig. 3k and l). Magnetic resonance imaging may provide information on skeletal changes, but has limited utility for evaluating lung parenchyma. Imaging MPS patients can be challenging. Sedation may be required for young or uncooperative patients, although complications may occur due to the high anaesthetic risk (Walker et al 2012).

### Assessment of respiratory function

Respiratory symptoms and hypoventilation may not be detected as many patients are inactive and since respiratory dysfunction is often poorly correlated with clinical appearance. Nevertheless, anticipation of respiratory problems and management at an early stage is important to maximize treatment outcome. Symptoms that require evaluation include progressive dyspnoea, reduced physical activity, exercise intolerance, wheezing or stridor, tachycardia and cyanosis.

Spirometry is the most frequent test utilized in MPS to assess the VC and the forced expiratory volume in 1 s (FEV_1_). Reduction in VC is suggestive of a restrictive abnormality while reduction in FEV_1_ (expressed as FEV_1_/VC) indicates airflow obstruction. Reduction in VC can reflect changes in lung compliance, chest wall compliance, and/or neuromuscular weakness. Reduced VC may also result from air trapping. Standard normal values for spirometry cannot be applied in MPS patients due to the disease-induced growth impairment and expressing VC as a % of normal is of limited value (Swiedler et al 2005). In addition, spirometry may not be possible in children who are too young to cooperate (< 5 years) and in patients with cognitive impairment. Despite its limitations, spirometry remains a valid clinical tool since all respiratory abnormalities that occur in MPS produce a decrease in VC. Spirometry has been commonly used for screening, to determine longitudinal disease progression and assess response to therapy.

Maximum voluntary ventilation (MVV) can be assessed as an alternative to spirometry. However, this test is physically demanding and can be difficult for MPS patients. Promising alternatives to assess for airflow obstruction include multiple breath inert gas washout and impulse oscillometry. These tests are performed during tidal breathing and may detect early airway disease (Aurora et al 2005; Oppenheimer et al 2007; Pasker et al 1996; Klug and Bisgaard 1996; Hellinckx et al 1998).

The degree of pulmonary restriction or hyperinflation can be measured using plethysmograhy, helium dilution or nitrogen washout. The diagnosis and management of parenchymal disease requires measurement of diffusion. This measurement is only reliable if the minimum volume inspired is 1.2 L and may not be possible in MPS patients with reduced lung capacity.

### Respiratory failure

Respiratory failure occurs from an imbalance between ventilation and metabolic rate. It can be identified by presence of hypercapnia on analysis of arterial blood gases. Since arterial blood gas analysis may not be available in the outpatient clinic, serum bicarbonate (HCO_3_^-^) concentration serves as a screening test since elevated values may indicate renal compensation for respiratory acidosis. If the serum [HCO_3_^-^] is ≥28 mEq/L, respiratory failure should be suspected and assessment of arterial blood gases should be considered (Mokhlesi et al 2007).

### Sleep studies

Sleep studies/polysomnograpy are used to diagnose and determine severity of respiratory sleep disorders. Since the incidence of SDB is high in MPS, polysomnography should be performed in all patients after diagnosis. Studies can be repeated based on presence of snoring, excessive daytime somnolence, development of respiratory failure and reduction in VC. Typical findings include recurrent OSA, tachycardia, decrease in arterial O_2_ saturation and recurrent arousal (Kamin 2008). It is important to distinguish OSA from sustained hypoventilation as these require different treatment approaches. Sustained nocturnal hypoventilation is suggested by oxygen desaturation in absence of OSA and can be confirmed by an increase in PCO_2_ during sleep, but this often carries over into wakefulness.

### Exercise tolerance testing

Exercise intolerance in MPS patients is generally measured using timed stair climbing or walk tests (e.g., 6- and 12-min walk tests [MWT]) (Harmatz et al 2008; Muenzer et al 2006; Wraith et al 2004). The 6-MWT is a practical test of submaximal functional capacity which mimics daily activities (e.g., American Thoracic Society 2002). Timed walk tests are used to assess functional status, monitor disease progression and response to interventions. The distance walked is generally the only variable that is evaluated. Valuable additional information could be obtained by measuring resting and end-exercise heart rate and arterial O_2_ saturation (e.g., American Thoracic Society 2002). Reference values for the 6-MWT exist (Enright and Sherrill 1998; Li et al 2007); however, applicability in MPS is unclear due to the associated cognitive and physical impediment.

Although not currently used, cardiopulmonary exercise testing might be valuable because of its wider diagnostic scope in terms of respiratory, cardio-circulatory and muscle-metabolic function (American Thoracic Society/American College of Chest Physicians 2003; Wasserman et al 2012). The symptom-limited ramp test provides a gradational stress spanning the entire tolerance range, with breath-by-breath measurement of ventilatory, gas-exchange and cardiovascular variables providing the substrate for analysing the causes of exercise intolerance, based on profiles of physiological system function.

## Management of airway problems in MPS

### Airway management

UA collapse at the pharyngeal and laryngeal level may occur due to rhinitis or adenoidal obstruction. Treatments may include nasal decongestions to control excessive mucus production, and steroids to reduce swelling. Adenoidectomy and/or tonsillectomy are also often required, and frequently performed before diagnosis of MPS. The procedure can be dangerous as surgeons may not anticipate potential anaesthetic and surgical complications and procedures should be performed by experienced personnel (Walker et al 2012; Simmons et al 2005). The operative field is frequently limited by thick and hard oropharyngeal tissue and UA scar tissue that may compromise the already small airway (Yeung et al 2009). In advanced cases, andenotonsillectomy can be inadequate or impossible. Moreover, any improvements achieved may be temporary because of progressive involvement of the oropharynx and trachea (Kamin 2008; Muhlebach et al 2011).

Patients with lower airway obstruction may use inhaled steroids to decrease airway inflammation. Because of instability of the tracheal and bronchial wall, patients may respond better to an anticholinergic bronchodilator agent than to a beta-agonist (Muhlebach et al 2011).

### SDB

The presence of SDB should prompt evaluation of the UA. Removal of tonsils and adenoids, if enlarged, may alleviate UA obstruction. SDB can also be managed by application of continuous positive airways pressure (CPAP), which delivers air at an elevated pressure using a mask that fits around the nose +/− mouth (Sullivan et al 1981; Rapoport et al 1982; Ginzburg et al 1990). The pressure acts as a dynamic stent and prevents airway collapse during inspiration. Nocturnal hypoventilation should be suspected if arterial O_2_ desaturation persists with CPAP or occurs in the absence of OSA (Berger et al 2001). In this case, non-invasive ventilator support systems (e.g., bilevel positive airway pressure) can be utilized and a backup respiratory rate can be added to prevent sustained hypoventilation. (Muhlebach et al 2011). Assisted ventilation may provide respiratory muscle rest and improve sleep quality, relieve nocturnal dyspnoea and lead to normalization of daytime blood gases. Supplemental O_2_ can be added if arterial O_2_ desaturation persists but should be used with caution as it may suppress ventilatory and arousal drives. Careful titration of the rate of O_2_ administration and monitoring of arterial CO_2_ levels are required to prevent O_2_-induced hypercapnia.

As compliance with CPAP and non-invasive ventilation is often low in MPS, compliance monitoring is imperative. In addition, leaks due to poor-fitting masks reduce treatment efficacy (Kamin 2008; Muhlebach et al 2011). Patients who adopt a sniff position may require specialized attention to ensure comfortable and adequate therapy. If properly used, CPAP and bilevel devices will improve breathing during sleep and provide relief of daytime somnolence and other consequences of SDB.

Tracheostomy may be required either when CPAP or non-invasive ventilation is ineffective or tracheomalacia and UA obstruction are present during wakefulness (Muhlebach et al 2011; Wold et al 2010). However, placement of the tracheostomy tube can be difficult and may be associated with stomal narrowing, granulation formation, infrastomal tracheal stenosis, wound infection, and tracheomalacia (Jeong et al 2006; Muhlebach et al 2011; Pelley et al 2007; Sims and Kempiners 2007). Problems may persist due to persistent airway collapse distal to the tip of the endotracheal tube (Pelley et al 2007) and patient preference for sleeping in the prone position. When the above therapies are ineffective, uncontrolled respiratory failure with cor pulmonale may develop and death may occur.

### Supportive therapies

Multiple supportive therapies are required to maintain optimal functional status. All patients should receive regular vaccinations, including pneumococcus and influenza vaccinations. Treatment of respiratory tract infections should be early and aggressive. Impairment in secretion clearance can be addressed by both manual and mechanical techniques. Inhaled bronchodilators can be used in conjunction with these airway clearance techniques. Bronchodilators and corticosteroids (inhaled and/or oral) may be useful if concurrent asthma is present. As restrictive pulmonary disease may be caused be spinal cord compression, pulmonary function may also improve after decompression surgery.

### Disease-targeting therapies

ERT is available for and beneficial in patients with MPS I, II, and VI. A positive impact has been demonstrated on 6- or 12-MWT and on pulmonary function (Clarke et al 2009; Harmatz et al 2004, 2005, 2006; Muenzer et al 2011, 2006; Okuyama et al 2010; Wraith et al 2004).

**Fig. 4:**
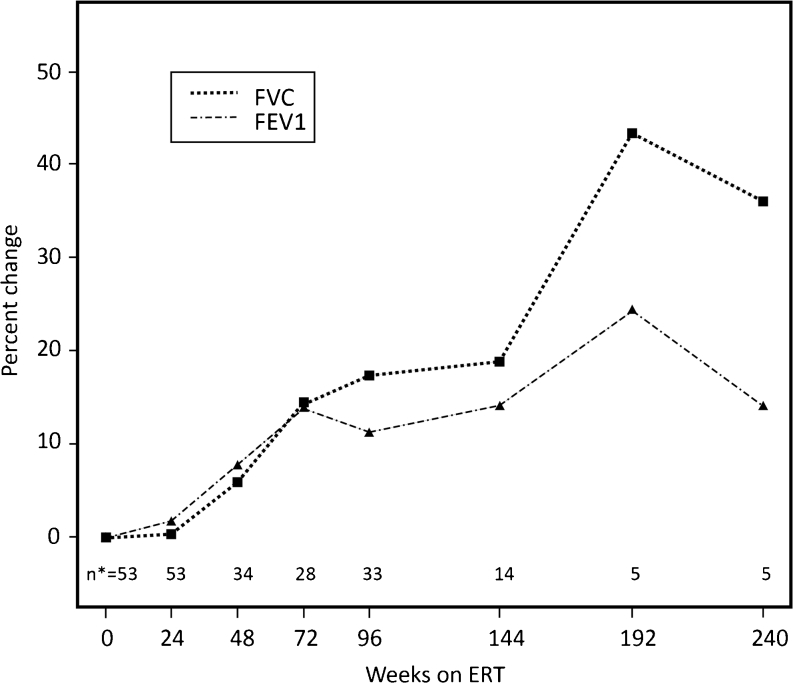
Changes from baseline in forced vital capacity (FVC) and forced expiratory volume in 1 s (FEV_1_) after initiation of enzyme replacement therapy (ERT). Results from a long-term extension study in patients with mucopolysaccharidosis VI (Harmatz et al 2010), reproduced with permission. n*: number of patients for whom data were available for that particular time point

An open-label extension study of ERT in MPS VI showed sustained improvement in walking distance for up to 240 weeks (Harmatz et al 2008). In addition, FEV_1_ and VC increased (11 % and 17 %, respectively) after 96 weeks of ERT with continued improvement thereafter (Fig. 4) (Harmatz et al 2010). Sustained improvement in MVV has also been demonstrated. These findings suggest that improved exercise tolerance in this population was related, at least partly, to improved pulmonary function.

The impact of ERT on pulmonary function should be interpreted in relationship to patient age. In younger children, increases in pulmonary function over time may (partly) be explained by growth. In line with this consideration, improvement in VC was only seen in patients with MPS II up to 18 years old (Muenzer et al 2011). In contrast, in MPS VI patients, (Harmatz et al 2010) increases in VC and FEV_1_ were observed in patients >12 years old despite minimal increases in height. In these patients, improvement in pulmonary function could only be explained by mechanisms other than growth, such as changes in bony structure, improvements in mobility, joint contractures or respiratory muscle function, shrinking of liver and spleen, or decreased GAG deposition in soft tissues. In adolescent patients, changes in pulmonary function may also be related to puberty.

Although haematopoietic stem cell transplantation (HSCT) is beneficial, and superior to ERT, for prevention of cognitive decline in young patients with MPS IH, effects in other MPS types tend to be poor (Rovelli 2008). Its use has been limited in the past by a high morbidity and mortality rate and the lack of matched donors, but this is changing with the increasing use of cord blood allowing a shorter interval between diagnosis and HSCT and a lower graft failure rate (Turbeville et al 2011; Prasad and Kurtzberg 2010). Most knowledge on the effect of HSCT on pulmonary function is based on case reports, some suggesting a positive impact (Krivit et al 1984; Wang et al 2008).

## Conclusions

The high prevalence of ENT and respiratory manifestations in MPS patients, their severity, and their appearance early in the disease course dictate that evaluation and follow-up of these problems is extremely important. Since asymptomatic patients may have severely impaired pulmonary function, pulmonary disease may go undetected for years without appropriate screening. However, the measurement of pulmonary function in MPS patients remains an important challenge due to the complexity of the disease and a lack of data on “usual pulmonary function” in these patients. Nevertheless, longitudinal follow-up of absolute values can be useful for monitoring the evolution of respiratory disease in a single patient. Current management guidelines for MPS I and MPS VI therefore recommend pulmonary function testing at diagnosis and every 6–12 months thereafter (Giugliani et al 2007; Muenzer et al 2009).

This review has addressed the pathophysiological mechanisms underlying respiratory manifestations in the different types of MPS. In addition, opportunities for enhanced evaluation of pulmonary function in these patients were highlighted. More sophisticated evaluation should help determine the mechanisms underlying the impact of ERT on exercise tolerance, VC, and FEV_1_.

## Electronic supplementary material

Below is the link to the electronic supplementary material.

Online supplementary material Video of upper airway and trachea obtained during fiberoptic bronchoscopy in a patient with MPS II. The posterior membraneous wall of the trachea is shown at the top of the image. Tracheal compression and distortion were noted. (MPG 4054 kb)
